# Supplementary material for: Neprilysin mediated cleavage of phospholamban dysregulates SERCA in heart failure
Source: bioRxiv. 2026 Jun 29:2026.06.23.732949. Preprint. [Version 1] doi: 10.64898/2026.06.23.732949 (PMC13344947; doi:10.64898/2026.06.23.732949)
Supplement: Supplement 2 [file media-2.pdf]

Major Resources Table

In order to allow validation and replication of experiments, all essential research materials listed in the Methods should be included in the Major Resources Table below. Authors are encouraged to use public repositories for protocols, data, code, and other materials and provide persistent identifiers and/or links to repositories when available. Authors may add or delete rows as needed.

Additional Species Used in This Study (if applicable)

Strain	Vendor or Source	Background Strain	Sex	Other Information (e.g. breeding scheme for F1, F2…if applicable)
Rat (Rattus norvegicus), neonatal, 0–1 day old	Charles River Laboratories	Sprague Dawley (outbred)	Not determined (neonatal pups, pooled)	N/A

Antibodies

Target antigen	Vendor or Source	Catalog #	Working concentration
Neprilysin / CD10 (NEP)	Abcam	ab256494	IF 1:100; WB 1:1,000
SERCA2 (ATP2A2)	Abcam	ab2817	IF: 1:500; WB 1:1000
Goat anti-rabbit IgG, AlexaFluor 488	Abcam	ab150077	1:1,000
Goat anti-rabbit/mouse IgG, AlexaFluor 568	Abcam	ab175471	1:1,000
Goat anti-rabbit IgG, IRDye 800CW	LI-COR Biosciences	926-32211	1:10,000
Phalloidin, AlexaFluor 647 (F-actin stain)	Cell Signaling Technology	8940	1:500
DAPI (nuclear stain; in Vectashield)	Vector Laboratories	H-1200	Stable formula

DNA/cDNA Clones

Clone Name	Sequence	Source / Repository	Persistent ID / URL
Unlabeled human neprilysin (MME)		Gift from H. Meyer, Universität Osnabrück	Schiemann et al. 2022 (ref 6)
mCer3–SERCA2a (human SERCA2a)		Robia laboratory	GenBank P16615; refs 42, 66, 67
YFP–PLB (human PLN)		Robia laboratory	UniProt P26678; ref 38
YFP–SLN (sarcolipin)		Robia laboratory	UniProt O00631; ref 38
YFP–ALN (another-regulin)		Robia laboratory	UniProt Q8WVX3; ref 38
YFP–ELN (endoregulin)		Robia laboratory	UniProt P0DI80; ref 38
YFP–MLN (myoregulin)		Robia laboratory	UniProt P0DMT0; ref 38
YFP–DWORF		Robia laboratory	UniProt P0DN84; ref 38
YFP–PLB I48X (C-terminal truncation)		Robia laboratory	Abrol et al. 2014 (ref 16)

DOI [to be added]

YFP–PLB V49A (cleavage-resistant)		Robia laboratory	ref 16 / this study
mCherry–NEP (N-terminal fusion, localization control)	This study	Created using ligation cloning from original construct (Schiemann et al. 2022 (ref 6))	
NEP–mCherry (C-terminal fusion, localization control)	This study	Created using ligation cloning from original construct (Schiemann et al. 2022 (ref 6))	

Cultured Cells

Name	Vendor or Source	Sex (F, M, or unknown)	Persistent ID / URL
HEK293T	Agilent (Santa Clara, CA)	Female	RRID:CVCL_0063
WTC hiPSC, ACTN2–mEGFP (clone 85)	Allen Cell Collection, via Coriell Institute	Male	AICS-0075-085
Neonatal rat ventricular myocytes (primary)	Isolated in-house (see Animals)	Undetermined	N/A

Data & Code Availability

Description	Source / Repository	Persistent ID / URL
Fiji / ImageJ (with custom macros)	NIH	RRID:SCR_002285
OriginPro 2025	OriginLab	RRID:SCR_014212
Proteome Discoverer 2.5 (Sequest HT)	Thermo Fisher Scientific	RRID:SCR_014477
Percolator (PSM validation)	Built into Proteome Discoverer	RRID:SCR_005040
Jalview v2.11.3.2	Jalview	RRID:SCR_006459
Clustal (alignment, via Jalview web service)	EMBL-EBI	RRID:SCR_001591
GO Functionome	Gene Ontology Consortium	https://functionome.geneontology.org/
MetaMorph	Molecular Devices	RRID:SCR_002368
ZEN Blue (joint deconvolution)	Carl Zeiss	RRID:SCR_013672
NIS-Elements	Nikon	RRID:SCR_014329
IonWizard	IonOptix	Cytosolver3
Xcalibur 4.5 / Tune 3.5	Thermo Fisher Scientific	RRID:SCR_014593

Other

Description	Source / Repository	Persistent ID / URL
Lipofectamine 3000 Transfection Kit	Invitrogen / Thermo Fisher	L3000015
Pierce BCA Protein Assay Kit	Thermo Fisher (Pierce)	23225
Fura-2 AM	Thermo Fisher	F1221
Revert Total Protein Stain	LI-COR Biosciences	926-11010
Intercept Blocking Buffer (PBS)	LI-COR Biosciences	927-70001
Vectashield mounting medium + DAPI	Vector Laboratories	H-1200
Matrigel	Corning	354277
mTeSR Plus	STEMCELL Technologies	100-0276
CHIR 99021	Tocris Bioscience	4423
IWP 2	Tocris Bioscience	3533
B-27 Supplement (± insulin)	Gibco / Thermo Fisher	17504044
Thiazovivin	STEMCELL Technologies	72252
Sodium L-lactate	Thermo Scientific Chemicals	L1450014
RPMI 1640, no glucose	Gibco / Thermo Fisher	11879020
10X TrypLE	Gibco / Thermo Fisher	A1217701

DOI [to be added]

KnockOut Serum Replacement	Gibco / Thermo Fisher	10828028
Saponin	Sigma-Aldrich	47036
Collagenase + 0.05% trypsin (NRVM digest)	C9891	C9891

Instrument	Vendor or Source	Model / Part #
Orbitrap Eclipse Tribrid MS + FAIMS Pro	Thermo Fisher Scientific	—
Vanquish Neo UHPLC	Thermo Fisher Scientific	—
EASY-Spray analytical column (25 cm)	Thermo Fisher Scientific	ES802A rev2
LSM 880 confocal / Airyscan	Carl Zeiss	—
Ti Eclipse 2 / Ti2-E inverted microscope	Nikon	—
Prime 95B sCMOS camera	Teledyne Photometrics	—
Spectra X light engine	Lumencor	—
c600 imaging system	Azure Biosystems	—
MyoPacer EP field stimulator + contractility system	IonOptix	—

Other — Databases, Deposited Data, and Human Specimens

Resource / Dataset	Source or Repository	Persistent ID / Accession
UniProt (Homo sapiens, UP000005640)	UniProt Consortium	RRID:SCR_002380
Bulk RNA-seq dataset (Figure 5)	GEO (NCBI)	GSE335813
Membrane-enrichment proteomics dataset (Figure 1)	ProteomeXchange / PRIDE	*pending*
Human LV tissue (NF n=5, DCM n=5; see Suppl. Table 1)	Loyola Cardiovascular Biorepository, Loyola University Chicago (de-identified specimens)	use of de-identified specimens for the present study was determined not to constitute human subjects research

DOI [to be added]
